# Higher number of items associated with significantly lower response rates in COS Delphi surveys

**DOI:** 10.1016/j.jclinepi.2018.12.010

**Published:** 2019-04

**Authors:** Elizabeth Gargon, Richard Crew, Girvan Burnside, Paula R. Williamson

**Affiliations:** a MRC North West Hub for Trials Methodology Research, Department of Biostatistics, University of Liverpool, Liverpool, UK; b Department of Biostatistics, University of Liverpool, Liverpool, UK

**Keywords:** Outcomes, Delphi, Research methodology, Core outcome set

## Abstract

**Objectives:**

The Delphi method is commonly used to achieve consensus in core outcome set (COS) development. It is important to try to maximize response rates to Delphi studies and minimize attrition rates and potential for bias. The factors that impact response rates in a Delphi study used for COS development are unknown. The objective of this study was to explore the impact of design characteristics on response rates in Delphi surveys within COS development.

**Methods:**

Published and ongoing studies that included Delphi to develop a COS were eligible. Second round voting response rates were analyzed, and multilevel linear regression was conducted to investigate whether design characteristics were associated with the response rate.

**Results:**

Thirty-one studies were included. Two characteristics were significantly associated with a lower response rate: larger panels and studies with more items included.

**Conclusion:**

COS developers should pay attention to methods when designing a COS development study; in particular, the size of the panels and the size of the list of outcomes. We identified other potential design characteristics that might influence response rates but were unable to explore them in this analysis. These should be reported in future reports to allow for further investigation.

**What is new?**

**Key findings**
• Multilevel linear regression was carried out to investigate what design characteristics were associated with response rates in studies that had included a Delphi study for core outcome set (COS) development. Two characteristics were significantly associated with a lower response rate. COS studies with larger panel sizes had significantly lower response rates, and studies that included a higher number of items had significantly lower response rates.
• Other characteristics, all previously identified as potentially influencing response rates, including item order, length of time between rounds, length of time each round is open, format of feedback, and details of reminders, were not reported in the Delphi study reports.
• The Delphi studies included in this study were predominantly e-Delphi studies. We were therefore unable to compare methods of delivery to investigate any impact this might have on the response rate.

**What this adds to what was known?**
• Previously, only one study has investigated the impact of design features on response rates, specifically the impact of item order within a Delphi survey. It showed that health professionals were less motivated to respond when clinical items appeared first. This is the first study to explore design characteristics beyond this.

**What is the implication and what should change now?**
• COS developers need to pay particular attention to panel size and number of items when designing a COS development study.
• Studies within studies to answer research questions identified in this study should be carried out, and will be an efficient and timely way to address the research uncertainties identified in this study.

## 1 Introduction

Problems with outcomes measured in trials and wider health research are well documented. Problems include outcome reporting bias [1], [2], inconsistency in measuring and reporting of outcomes [3], and relevance to patients [4]. These problems can lead to the use of ineffective or even harmful interventions and to the waste of health care resources that are already limited [5]. These problems are being addressed by the development and application of core outcome set (COS), the minimum agreed set of outcomes that should be measured and reported in all trials for a specific clinical area [6]. COS are also applicable in other settings such as for use in systematic reviews and routine care or audit. The focus of this article is COS developed for research studies. When developing a COS, it is typical to first gain agreement about “what” to measure, with decisions about “how and when” to measure these outcomes later in the process [7]. This article examines studies that have gained agreement about “what” should be measured.

Systematic reviews of COS demonstrate the growing number of COS developed for research [8], [9], [10], [11] and an increase in the use of Delphi in their development [10]. This suggests that developers are increasingly adopting a more structured approach to COS development, which in turn, has prompted the publication of the Core Outcome Measures in Effectiveness Trials Handbook Version 1.0 [7], to bring together accumulating methodological work in this area and offer recommendations for COS development.

A systematic review of studies that used the Delphi technique to determine which outcomes to measure in clinical trials concluded that there was variability in both methodology and reporting [12]. This resulted in recommendations to improve the quality of studies that use the Delphi process for determining outcomes to use in clinical trials, including the recommendation that patients and clinicians be involved, researchers and facilitators avoid imposing their views on participants, and attrition of participants be minimized. Attrition of participants could mean that people with minority opinions drop out of the Delphi study, leading to an overestimation of consensus. The validity of the results will ultimately be affected by response rates [13]. It is therefore important to try to maximize response rates to a Delphi study, minimizing attrition rates and therefore any potential attrition bias. Furthermore, qualitative interviews with COS developers highlighted response rates in Delphi studies for COS development as a priority area for further research and guidance [14].

In the context of COS development, we are aware of only one empirical research study investigating the impact of design features on response rates. Brookes et al. used a parallel randomized controlled trial design nested within a Delphi survey for COS development, to explore the impact of item order within a Delphi on the response rates [15]. The impact of item order within a Delphi survey showed that item order mattered, and health professionals appeared to have been less motivated to respond when clinical items appeared first. Response rates in Delphi studies for COS development have not previously been explored beyond this, and so the design characteristics that impact response rates in a Delphi study used for COS development are unknown.

## 2 Aims

The objective of this study was to explore the potential impact of different design characteristics on response rates in Delphi surveys within COS development projects. The following hypotheses were considered:

1. The number of rounds will affect the response rate, with the expectation that response will decrease as the number of rounds in a Delphi study increases.
2. The number of items included in a round of voting will affect the response rate, with the expectation that Delphi response rates will be lower in studies that have included a higher number of items.
3. The size of the panel will affect the response rate, with smaller panel sizes having a higher response rate.

Other characteristics, including single vs. multidisciplinary panels; international participation; format of feedback; length of time between rounds; length of time of each round; whether reminders were sent between rounds; mode of delivery; and acute vs. chronic health conditions, were also considered.

## 3 Methods

### 3.1 Eligible studies

#### 3.1.1 Inclusion criteria

A previous systematic review, with subsequent updates, of COS have identified 259 published studies up to and including December 2016 [8], [9], [10], [11]. The methods of those reviews are reported in accordance with PRISMA guidelines in the original publication [8]. Methodology reporting of older studies was poor; this study was therefore limited to more recently published studies in the hope that they would report relevant information and data. Studies from the three update reviews [9], [10], [11] that included Delphi in their methods to develop a COS were eligible for inclusion in this study.

Ongoing studies that have used DelphiManager in the development of a COS, and that had completed the second round of voting in the study, were also eligible for inclusion in this study. These were identified via the DelphiManager software developer (R.C.). DelphiManager is a web-based system designed to facilitate the building and management of e-Delphi surveys, and includes functionality allowing the researcher to email participants regarding missing responses, and view the accumulating response rates for rounds two and beyond. Permission was sought from each of the ongoing COS developers to include their data in this study.

#### 3.1.2 Exclusion criteria

Delphi studies that did not report response data, or that did not report both a numerator and denominator for the response rate (e.g., only reporting total percentage response rates), were excluded. Studies with only one round of voting were also excluded from this study. Studies that had not yet completed the second round of voting were excluded from this study.

### 3.2 Data extraction

First round response rates were described using varying denominators, including the number of participants invited and the number of participants who agreed to participate, making it difficult to combine, compare, and contrast. We therefore analyzed the response rates in the second rounds, where the denominator is the number of participants invited to the second round of voting. This was not necessarily the round named “round 2” in the reports. Furthermore, some studies included an open first round for generating the list of outcomes therefore including a nonvoting round. This was therefore not counted as a first round for the purposes of this study. Hereafter, when using the term “response rate,” we are therefore referring to response rate in the second round. All ongoing study data included in the analysis and write-up of this study has been pseudoanonymized, with the removal of identifiable features, such as place and person names. Details necessary for analysis (e.g., clinical area) were retained.

The following information was extracted for each included Delphi study:

1. Study details including
  a. Surname of first author
  b. Year of publication
  c. Disease area/name
2. Design
  a. Mode of delivery used
  b. Recruitment method
  c. Participant contact method
  d. Whether a reminder was sent between rounds (and method of reminder)
  e. Number of rounds
  f. Number of items included in each round
  g. Number of panels
  h. Format of feedback
  i. Length of time between each round
3. Participants
  a. Stakeholder groups
  b. Countries
4. Response rate
  a. Number of participants overall who completed each round
  b. Number of participants overall invited to complete each round
  c. Number of participants by panel who completed each round
  d. Number of participants by panel invited to complete each round

Authors were contacted by email and asked to provide any missing data where possible.

### 3.3 Analysis

Multilevel linear regression was carried out to investigate whether Delphi design characteristics were associated with second round response rate. The models were fitted to reflect the panel structure within studies. The response variable in the model was the percentage response rate in the second round for each panel. Independent variables included for each panel were panel composition (mixed discipline/single discipline), size of panel invited to the second round, and whether the panel was international or based in a single country. Independent variables included for each study were number of rounds in the study and number of items included in the second round. An initial model was fitted with random intercept. The effect of adding random slopes for each independent variable was explored by comparing the model fit using the Akaike Information Criterion. Models were fitted using PROC MIXED in SAS software version 9.4.

Third and fourth round response rate data were not analyzed due to the small sample size included in this study.

## 4 Results

Thirty studies from the systematic review updates [9], [10], [11] used Delphi methodology. Six studies did not report response rates, did not report response rate data that were useable, or only included one round of voting, and were consequently excluded from this study.

Eight ongoing COS studies (as of February 2018) had completed the second round of voting using DelphiManager. Seven gave permission for their study data to be included and were therefore included in this study. The declined study author cited complex governance procedures, combined with personal circumstances, as the reason for not contributing to this study.

These results therefore pertain to 31 studies, 24 published and seven ongoing studies that had used Delphi in the process of developing a COS.

A descriptive summary of key characteristics is provided in Table 1.

**Table 1:** A descriptive summary of key design characteristics

Study characteristics (*n* = 31)	*n* (%)	Panel characteristics (*n* = 72)	*n* (%)
Number of rounds		Panel size	
2	13 (42)	1–50	44 (61)
3	16 (52)	51–100	16 (22)
4	2 (6)	101–150	4 (6)
Method of Delivery		151–200	3 (4)
e-Delphi	25^a^ (81)	201–250	2 (3)
Post	3 (10)	251–300	2 (3)
e-Delphi (clinician) and post (patient)	2 (6)	301–350	0 (0)
Not reported	1 (3)	351–400	1 (1)
Number of panels		Panel composition	
1	14 (45)	Clinical experts (multidisciplinary)	20 (28)
2	8 (26)	Clinical experts (single discipline)	19 (26)
3	2 (6)	Patient and public representatives	18 (25)
4	1 (3)	Mixed	8 (11)
5	3 (10)	Researchers	5 (7)
6	1 (3)	Funder	1 (1)
7	1 (3)	Commercial representative	1 (1)
8	1 (3)	Participant countries	
Number of items		National (one country only)	25 (35)
1-50	17 (55)	International (more than one country)	47 (65)
51-100	10 (32)		
101-150	3 (10)		
Not reported	1 (3)		
Reminders sent between rounds			
Yes	19 (61)		
No/not known	12 (39)		

a Paper version available for patients on request in two studies.

### 4.1 Overall response rates

Overall sample sizes ranged from 9 to 678 (median 110). Response rates for the second round of voting ranged from 45% to 100%. These data, per study, are provided in Table 2. The overall response rate is typically 80% or higher, with only four studies where the overall response rate in the second round of voting is below this at 45%, 50%, 52%, and 64%. Email reminders were sent between first and second rounds of voting in nineteen studies to encourage participation and increase response rates.

**Table 2:** Study details

Study	Number of rounds	Method of delivery	Number of panels (panel composition)	Number of items included in second round of voting	Second round of voting total response *N* completed the round *N* invited to the round	Second round of voting total response (%)
Buch (2014)	2	e-Delphi	1 (Mixed)	26	21/21	100
Currie (2015)	2	e-Delphi	1 (Clinical experts: multidisciplinary)	32	33/33	100
Major (2016)	3^a^	e-Delphi	1 (Mixed)	87	10/10^a^	100
Ward (2014)	3^a^	e-Delphi	1 (Experts in Yoga)	31	36/37^a^	97
Wylde (2014)	3	Clinician panel e-Delphi Patient panel paper by post	2 (Patient and public; clinical experts: multidisciplinary)	33	102/110	93
Gerritsen (2016)	2	e-Delphi (but paper for patients available on request)	2 (Patient and public; clinical experts: multidisciplinary)	49	208/228	91
Smelt (2014)	3	e-Delphi	1 (patient and public)	36	152/169	90
Balakrishnan (2015)	3	Not reported	1 (Clinical experts: multidisciplinary)	64	8/9	89
van 't Hooft (2015)	2	e-Delphi	5 (Patient and public; researchers; clinical experts: single discipline ×2; researcher)	31	174/195	89
Helliwell (2016)	3	e-Delphi (but paper for patients available on request)^e^	2 (Patient and public; clinical experts: multidisciplinary^e^)	19 clinician panel/23 patient panel^e^	101/115	88
Milman (2017)	3^a^	e-Delphi	1 (Clinical experts: multidisciplinary)	77^e^	36/41^a^	88
Ismail (2016)	2	e-Delphi	1 (Mixed)	51^b^	56/65	86
Harman (2015)	3	e-Delphi	6 (Clinical experts: single discipline ×6)^d^	47	85/99	86
Haeusler (2015)	4	e-Delphi	1 (Mixed)	29^c^	37/43	86
Potter (2015)	2	Post	2 (Patient and public; clinical experts: multidisciplinary)	148	259/303	86
Eleftheriadou (2015)	3	e-Delphi	3 (Patient and public; mixed; clinical experts: single discipline)	8	87/101	86
McNair (2016)	2	Post	2 (Patient and public; clinical experts: multidisciplinary)	45	165/195	85
Smith (2014)	2	e-Delphi	1 (Clinical experts: single discipline)	Not reported	10/12	83
Coulman (2016)	3	Clinician panel paper by post OR e-Delphi Patient panel paper by post	2 (Patient and public; clinical experts: multidisciplinary)	130	200/246	81
Janssens (2014)	4^a^	Post	1 (Clinical experts: multidisciplinary)	22	227/285^a^	80
Fair (2016)	2	e-Delphi	1 (Mixed)	13	93/117	80
Al Wattar (2017)	3	e-Delphi	3 (Clinical experts: multidisciplinary ×2; Clinical experts: single discipline)	48	48/75	64
Audigé (2016)	3	e-Delphi	1 (Clinical experts: single discipline)	9	69/132	52
Chiarotto (2015)	3	e-Delphi	1 (Mixed)	51	130/261	50
DM1	2	e-Delphi	7 (Clinical experts: multidisciplinary ×2; clinical experts: single discipline ×3; patient and public × 1; mixed × 1)	100	141/205	69
DM2	3	e-Delphi	2 (Patient and public; clinical experts: multidisciplinary)	57	86/93	92
DM3	2	e-Delphi	5 (Patient and public × 2; clinical experts: multidisciplinary; experts: single discipline; researcher)	79	36/51	71
DM4	3	e-Delphi	6 (Patient and public × 2; clinical experts: multidisciplinary; experts: single discipline ×2; researcher)	114	187/416	45
DM5	2	e-Delphi	2 (Patient and public; clinical experts: multidisciplinary)	78	141/169	83
DM6	3	e-Delphi	5 (Patient and public; Clinical experts: multidisciplinary; researcher; funder; commercial representative)	68	581/678	86
DM7	2	e-Delphi	4 (Patient and public; clinical experts: multidisciplinary; researcher, funder)	36	74/76	97

a Round one was for generating the list of outcomes, so this is the response rate for round three (R3), as this is equivalent to the second round of voting in the other Delphi studies.

b 7 outcomes and 44 measures.

c 13 variables and 16 outcomes.

d The study had eight panels in R1: one was not invited to participate beyond R1, and one was combined with another group after the first round of voting. These two panels are therefore excluded here.

e Confirmed/provided through personal communication with the author.

#### 4.1.1 Number of rounds

Thirteen studies included two rounds, sixteen studies included three rounds, and two studies included four rounds (Table 2). Studies that included two rounds had a range of response rates between 69% and 100%. Studies that included three rounds reported response rates between 45% and 93%. The two studies with four rounds reported response rates of 80% and 86%, respectively. Number of rounds was not significantly associated with response rate (*P*-value 0.634) (Table 3).

**Table 3:** Results of the multilevel linear regression analysis

Variable	Coefficient	95% Confidence interval	*P*-value
Panel level variables			
Panel composition (mixed vs. single)	1.44	−4.15, 7.03	0.598
Size of panel	−0.08	−0.15, −0.01	0.035
International vs. national	0.53	−6.82, 7.89	0.882
Study level variables			
Number of rounds	−1.57	−8.31, 5.17	0.634
Number of items	−0.14	−0.25, −0.03	0.017

#### 4.1.2 Number of items

Three studies did not report the number of items included per round, but this was provided for two studies when authors were contacted by email. This analysis therefore relates to 30 studies. The number of items included in the second round of voting ranged from 8 to 148, and the number of items per study is reported in Table 2.

**Fig. 1:**
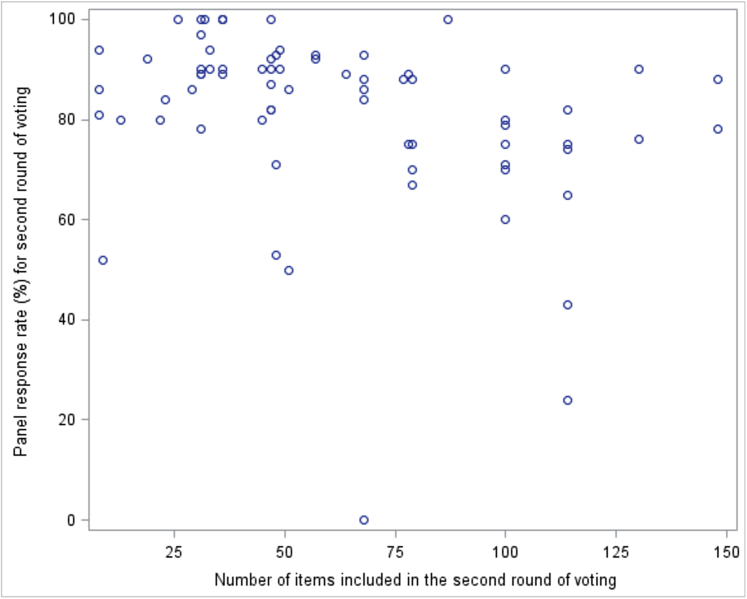
Is there an association between the number of items included in the second round of voting and the response rate in the second round of voting?

The multilevel linear regression analysis demonstrated a significant association between number of items and response rate in the second round (*P*-value 0.017) (Table 3), where studies with more items included in the second round had significantly lower response rates. The coefficient for number of items is −0.14, so for every 10 additional items included in the round, the estimated response rate drops by 1.4 percentage points. The association between number of items and response rate is displayed graphically in Fig. 1.

### 4.2 Response rates by panel

The number of stakeholder panels per study ranged between one and eight. A summary is provided in Table 1, and by study in Table 2. In the study that had eight panels, one of the panels was not invited to participate beyond round 1 (R1), and one was combined with another group after the first round of voting. These two panels are therefore excluded from further analysis, and this study is included as having six panels for the purpose of this study. Two panels were excluded from DelphiManager studies; one of the panels was not invited to participate beyond R1 and so is therefore excluded from further analysis, and another panel was excluded from the final analysis because it had a single member that did not respond to the second round, giving a 0% response rate. The analysis by panel therefore relates to 72 panels (summarized in Table 4, described in more detail in Appendix 1).

**Table 4:** A summary of panels (see Appendix 1 for description)

Stakeholder group	Number of panels	Range of response rates (%)	Range of number of items
Clinical experts (single discipline)	19	52–100	8–114^a^
Clinical experts (multidisciplinary)	20	53–100	22–148
Patient and public representatives	18	24–94	8–148
Mixed	8	50–100	8–100
Other (single panels)^b^	7	74–100	31–114
Total	72		

a Not reported for one panel.

b Includes 5 researcher panels, 1 funder panel, and 1 commercial representative panel.

Studies that included only one panel had a response rate range of between 50% and 100%. Studies with two panels had panel response rates between 75% and 94%. Studies with three panels had panel response rates between 53% and 94%, and finally, studies with four or more panels had panel response rates between 24% and 100%.

In all but one of the published studies with multiple panels, feedback in the second round of voting was provided separately for each panel (i.e., the different stakeholder groups). In the one exception, feedback was provided at the start of the second round of voting as the mean scores and standard deviations for each outcome; they then provided scores by each stakeholder group at the start of the third round of voting. In all but one of the Delphi Manager studies, feedback in the second round of voting was provided separately for each panel. In the remaining study, it was provided as combined percentage distribution across all panels.

#### 4.2.1 Panel size

**Fig. 2:**
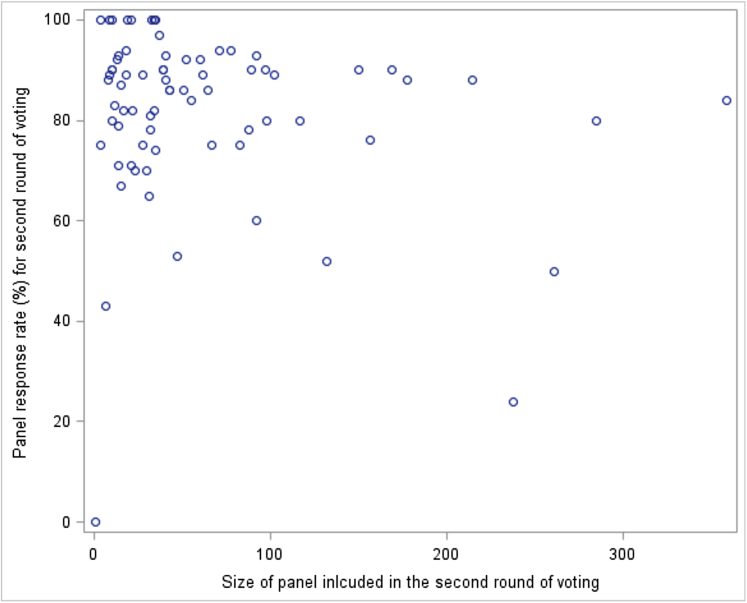
Is there an association between the size of the panel going into the second round of voting, the response rate in the second round of voting, and the panel composition?

The only independent variable where allowing random slopes improved model fit was the size of the panel. Random slopes for this variable were added to the final model. The multilevel linear regression analysis demonstrated a significant association between the size of the panel and response rate (*P*-value 0.035) (Table 3), where larger panels had significantly lower response rates. The coefficient for the panel size is −0.08; that is, that the estimated response rate drops by 0.08 of a percentage point on average for every additional member. Therefore, as an example, for an additional 10 members, estimated response rate dropped by approximately 0.8 of a percentage point, and for an additional 50 members, it dropped by four percentage points. Fig. 2 shows the association between size of panel and response rate.

#### 4.2.2 Panel composition

The range of response rates in the second round for different stakeholder group panels was similar for each panel composition. If panels are considered as mixed (including those described here as multidisciplinary clinical experts and mixed) compared to those of a single discipline (including here the descriptor clinical experts' single discipline, other, and patient and public representatives), then the response rates are also similar. Panel composition (mixed vs. single) was not significantly associated with response rate (*P*-value 0.598) (Table 3).

#### 4.2.3 Participant countries

25/72 panels (35%) only included participants from one country (national panels). National panels had response rates of 53%–100%. The remaining 47 (65%) panels included participants from more than one country (international panels), between 3 and 25 countries in the 17 that reported this information (one from correspondence with author). International panels had response rates of between 24% and 100%. Participant countries (national vs. international) were not significantly associated with the response rate (*P*-value 0.882) (Table 3).

### 4.3 Other characteristics

Insufficient data were reported on the format of feedback provided, the length of time that each round was open, and the length of time between rounds. We were therefore unable to consider these characteristics in the analysis. The Delphi studies included in this study were predominantly e-Delphi studies, that is, delivered electronically, and therefore, we were unable to compare against other modes of delivery such as postal. Finally, the COS in these Delphi studies were developed principally for chronic conditions, again making it impossible to make a comparison against COS for acute conditions.

## 5 Discussion

Multilevel linear regression was carried out to investigate whether design characteristics were associated with second round response rates in studies that had included a Delphi survey as part of COS development. Two characteristics were significantly associated with a lower response rate: larger size of panels and studies with more items included in the second round.

Studies that included a higher number of items had significantly lower response rates in the second round. It has previously been shown that odds of response increase for shorter surveys [16]. Use of a shorter list might minimize nonresponse, but this would need to be traded-off against the need for the list of outcomes to be comprehensive. While it might be regarded as more comprehensive to retain outcomes through the rounds, retaining all outcomes through all rounds on a large list may be burdensome to participants and increase attrition between rounds [7]. If the decision is made to reduce the number of items between rounds to lessen the burden on participants, this should be clearly stated a priori and the criteria for doing so defined in advance to avoid bias [17].

**Fig. 3:**
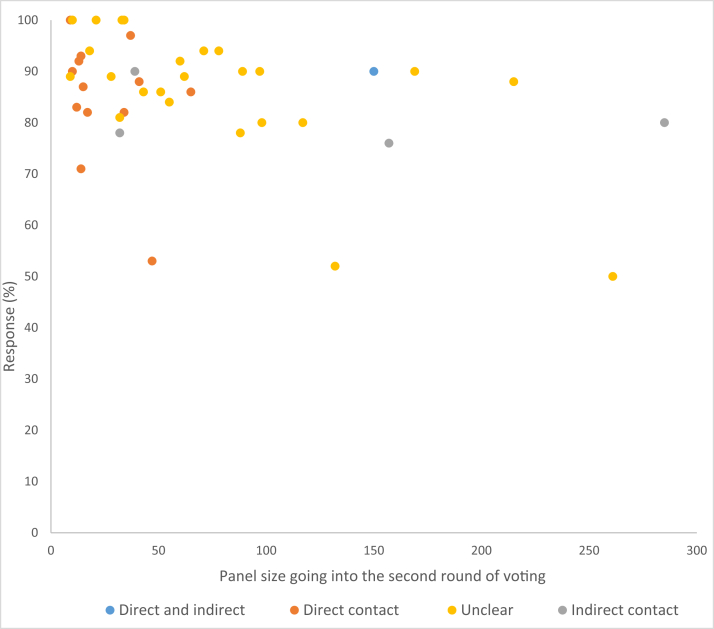
Panel size and method of contact.

COS studies with smaller panel sizes had significantly better response rates in the second round. The method of recruitment and participant contact at the start of the study might explain why smaller panels have higher response rates. Smaller panels were contacted directly by the study team (this information is only available for published studies, see Fig. 3) compared to studies with larger panels that utilized an indirect approach of contact such as a website call through a charity or professional organization or the method was unclear. Adopting a personalized approach has been suggested to increase odds of response for surveys more generally [16], and it would seem that this is also true for Delphi surveys in COS development.

A Delphi study must consist of a minimum of two rounds (at least one round of feedback) to be considered a Delphi survey [7]. We hypothesized that the number of rounds would have an inverse association with response, as COS developers have previously expressed concern around retaining participants over the course of a study [14]. The number of rounds was not associated with second round response rates in this study. Furthermore, because the first round of voting was reported with varying denominators, we needed to analyze the second round of voting in this study. Participants in these studies were already committed to the study and so are likely to explain the high levels of response rates seen in most COS Delphi studies included in this analysis.

Panel composition was not associated with response rate when comparing panels of heterogeneous participants with panels of experts from one particular stakeholder group. We hypothesized that single discipline panels would have higher response rates because the outcomes rated by a homogenous group might be more or less relevant to that particular group, and so individual participants might identify more with the outcomes, and therefore more likely to respond. However, with the exception of one study that had some differences in the outcomes being rated between panels, all panels within studies were presented with the same list of outcomes, which could explain why no difference was detected. Interviews are currently underway to explore participant perspectives on how relevant the list of outcomes was for them and whether this affected their behavior and decision on whether to complete the Delphi or not (personal communication).

We hypothesized that response rates would decrease with international participation but did not find an association between international panels and lower response rates. The number of countries included in most international studies was not reported, so we were not able to explore this. Although this study did not find a significant association, the challenges of working internationally have been highlighted previously, including the resources required for international participation [7], [14].

Sending a reminder in-between rounds has previously reported increasing odds of survey response by more than a quarter [16]. However, there are many underlying complexities in sending reminders, including whether it was an a priori or ad hoc decision, timing of reminder, and length of time the round remains open following the reminder. COS study reports do not include such in-depth information about reminders, which meant we were unable to include this in the analysis in this study. Furthermore, the inclusion of whether a reminder was sent or not was not deemed reliable enough to include as a simple variable as reminder sent or not, as it was plausible that this might not have been reported for all studies where response rates were already regarded as high. A recent COS study asked participants about their experiences of participating in a COS Delphi, and it was concluded that participants did not find it bothersome to receive reminders to encourage timely voting [18]. Furthermore, another study has recently highlighted methods that worked well in relation to recruitment and retention, including the sending of reminders [19]. Reminders should continue to be sent in an attempt to maximize response.

We identified other potential characteristics that might influence response rates, including the mode of delivery of the Delphi survey. Research has previously shown that internet-based questionnaires are associated with lower response rates than postal [20]. Almost all the studies included in this study were administered online, so we were unable to explore any potential differences between postal and e-Delphi surveys. Other factors, all previously identified as potentially influencing response rates [7], [15], including item order, length of time between rounds, length of time each round is open, format of feedback, and the aforementioned reminder complexities, were not reported in the Delphi study reports. These details should be included in future reports to allow comparisons to be made. Furthermore, studies within studies to answer research questions should be carried out and will be an efficient and timely way to address the research uncertainties identified in this study [21]. The final characteristic that we considered in relation to response rates in Delphi studies for COS development was whether the COS was developed for an acute or chronic disease population. Research in clinical trial recruitment has shown that in practice, recruitment and retention rates vary depending on this [22]. We were unable to analyze this for the Delphi COS studies included in this study because they were predominantly developed for chronic conditions.

This is the first study to investigate the association of a range of characteristics to response rates in Delphi studies for COS development. By including ongoing COS as well as published, the conclusions drawn are current and likely to remain relevant to inform COS development for the foreseeable future. Ongoing studies were identified through the use of DelphiManager only; we therefore do not know the extent to which our findings might be relevant to ongoing COS Delphi studies using other software, although it is not expected that these studies would have any differing characteristics. The use of multilevel linear regression allows multiple panels within a single study to be included in the analysis, without the assumption that individual panels within a study are independent observations. It should be acknowledged that this was an exploratory analysis with a relatively small sample size, and that, these results should be interpreted as indications of potential associations and not definitive causal relationships. A larger study is needed to confirm these findings.

In summary, this analysis showed that larger panels, and studies with more items included in the round, had significantly lower response rates. COS developers should pay particular attention to these characteristics when designing a COS development study. Suitable early planning is essential to optimize response rates in the Delphi process.

## Supplementary data

Data Profile
