# Supplementary material for: Higher number of items associated with significantly lower response rates in COS Delphi surveys
Source: J Clin Epidemiol. 2019 Apr;108:110–20. doi: 10.1016/j.jclinepi.2018.12.010 (PMC6438267; doi:10.1016/j.jclinepi.2018.12.010)
Supplement: Appendix [file mmc2.docx]

**Appendix 1: A description by panel (n=72) of study recruitment methods and participant contact methods used in Delphi studies**

| **Study** | **Panel** | **Size of second round voting panel** | **Second round of voting panel response %** | **Study recruitment methods** | **Participant contact method** | **Number of countries (Counties)** |
| --- | --- | --- | --- | --- | --- | --- |
| Harman (2015) | Clinical experts (single discipline) | 9 | 100 | Clinical centres | Individual participants emailed directly | 1 (UK) |
| Harman (2015) | Clinical experts (single discipline) | 10 | 90 | Clinical centres | Individual participants emailed directly | 1 (UK) |
| Harman (2015) | Clinical experts (single discipline) | 13 | 92 | Clinical centres | Individual participants emailed directly | 1 (UK) |
| Harman (2015) | Clinical experts (single discipline) | 15 | 87 | Clinical centres | Individual participants emailed directly | 1 (UK) |
| Harman (2015) | Clinical experts (single discipline) | 17 | 82 | Clinical centres | Individual participants emailed directly | 1 (UK) |
| Harman (2015) | Clinical experts (single discipline) | 34 | 82 | Clinical centres | Individual participants emailed directly | 1 (UK) |
| van ʼt Hooft (2015) | Clinical experts (single discipline) | 62 | 89 | Professional organisations | A formal written invitation was e-mailed to all members | 25 (not stated) |
| van ʼt Hooft (2015) | Clinical experts (single discipline) | 28 | 89 | Clinical centres  Professional organisations | A formal written invitation was e-mailed to all members of organisations | 25 (not stated) |
| van ʼt Hooft (2015) | Clinical experts (single discipline) | 34 | 100 | Professional organisations | A formal written invitation was e-mailed to all members | 25 (not stated) |
| Al Wattar (2017) | Clinical experts (single discipline) | 14 | 93 | Investigators’ email database | Direct email | 1 (UK) |
| Eleftheriadou (2015) | Clinical experts (single discipline) | 51 | 86 | Professional organisations | Email invite sent | 24 overall – not broken down to panel (Algeria, Australia, Austria, Bahrain, Belgium, Brazil, Canada,  Colombia, Egypt, France, Germany, Greece, India,  Italy, Japan, Mexico, Morocco, the Netherlands, Saudi  Arabia, Spain, Taiwan, Tunisia, United Arab Emirates,  United Kingdom and United States of America.) |
| Audigé (2016) | Clinical experts (single discipline) | 132 | 52 | Personal knowledge of experts  Professional organisation/society | Invited by email | Numbers only reported for R3 (international) |
| Smith (2014) | Clinical experts (single discipline) | 12 | 83 | Publications  Currently undertaking research | Invited by email | 10 (China, England, Australia, France, Italy, Spain, Canada, France, Holland, Japan)  *France included in the list twice* |
| Ward (2014) | Clinical experts (single discipline) | 37 | 97 | Publications Experts known to participants | Personalised emails | 6 (US, Turkey, Brazil, UK, India, Sri Lanka) |
| DM1 | Clinical experts (single discipline) | 14 | 79 | Not available | Not available | International*^~^* |
| DM1 | Clinical experts (single discipline) | 92 | 60 | Not available | Not available | International*^~^* |
| DM1 | Clinical experts (single discipline) | 10 | 80 | Not available | Not available | International*^~^* |
| DM4 | Clinical experts (single discipline) | 83 | 75 | Not available | Not available | International*^~^* |
| DM4 | Clinical experts (single discipline) | 31 | 65 | Not available | Not available | International*^~^* |
| Al Wattar (2017) | Clinical experts (multidisciplinary) | 47 | 53 | Investigators’ email database. | Direct email | 1 (UK) |
| Al Wattar (2017) | Clinical experts (multidisciplinary) | 14 | 71 | Investigators’ email database. | Direct email | 1 (UK) |
| Balakrishnan (2015) | Clinical experts (multidisciplinary) | 9 | 89 | Members of a taskforce/working group | Not clear | Not reported |
| Wylde (2014) | Clinical experts (multidisciplinary) | 39 | 90 | Professional organisations  Previous research participation Publications Experts known to participants | Organizational gatekeepers were asked to disseminate study information via e-mail. | 3 (UK, Canada, Australia) |
| Currie (2015) | Clinical experts (multidisciplinary) | 33 | 100 | Publications Known to experts | Invited (assume by email as first round was sent by email) | 11 (not stated) |
| Janssens (2014) | Clinical experts (multidisciplinary) | 285 | 80 | Professional societies  Child Development Teams | Professional societies forwarded invitations to their members  Participants registered to take part using an online form | 1 (UK) |
| Gerritsen (2016) | Clinical experts (multidisciplinary) | 78 | 94 | Professional organisations | Electronic invitation | 1 (The Netherlands) |
| Potter (2015) | Clinical experts (multidisciplinary) | 88 | 78 | Previous research participation Publications | Invitation letter by post | 1 (UK) |
| Coulman (2016) | Clinical experts (multidisciplinary) | 157 | 76 | Professional organisations  Previous research participation | Emailed/post by their society | 3 (UK, Republic of Ireland, Belgium)  *5 participants not specified* |
| McNair (2016) | Clinical experts (multidisciplinary) | 98 | 80 | Clinical centres | Participants were approached by post | 1 (UK) |
| Helliwell (2016) | Clinical experts (multidisciplinary) | 60 | 92 | Not clear | Not clear | Not reported (international) |
| Milman (2017) | Clinical experts (multidisciplinary) | 41 | 88 | Professional organisations | Email invite sent | 18 (Canada, Mexico, US, Brazil, Chile, Peru, Denmark, Germany, Iceland, Italy, Netherlands, Spain, Sweden, UK, Japan, Turkey, Australia, New Zealand) |
| DM1 | Clinical experts (multidisciplinary) | 28 | 75 | Not available | Not available | International*^~^* |
| DM1 | Clinical experts (multidisciplinary) | 10 | 90 | Not available | Not available | International*^~^* |
| DM2 | Clinical experts (multidisciplinary) | 52 | 92 | Not available | Not available | International*^~^* |
| DM3 | Clinical experts (multidisciplinary) | 23 | 70 | Not available | Not available | 1 (UK) *^~^* |
| DM4 | Clinical experts (multidisciplinary) | 22 | 82 | Not available | Not available | International*^~^* |
| DM5 | Clinical experts (multidisciplinary) | 102 | 89 | Not available | Not available | International*^~^* |
| DM6 | Clinical experts (multidisciplinary) | 178 | 88 | Not available | Not available | International*^~^* |
| DM7 | Clinical experts (multidisciplinary) | 19 | 100 | Not available | Not available | International*^~^* |
| Wylde (2014) | Patient and public representatives | 71 | 94 | Previous research participation | Sent a study pack (post) | 1 (UK) |
| Coulman (2016) | Patient and public representatives | 89 | 90 | Clinical centres | Invited to participate (I think post) | 1 (UK) |
| McNair (2016) | Patient and public representatives | 97 | 90 | Clinical centres | Participants were approached by post | 1 (UK) |
| Gerritsen (2016) | Patient and public representatives | 150 | 90 | Clinical centres Patient organisations | Clinical centre: all patients meeting the criteria were contacted by telephone and asked whether they would be willing to participate in the survey.  Patient organisation: website call | 1 (The Netherlands) |
| Smelt (2014) | Patient and public representatives | 169 | 90 | Patient database | Invitation by email | 1 (The Netherlands) |
| Potter (2015) | Patient and public representatives | 215 | 88 | Clinical centres | Invitation letter by post | 1 (UK) |
| van ʼt Hooft (2015) | Patient and public representatives | 32 | 78 | Patient organisations | Patient organisations approached members | Not reported (international) |
| Eleftheriadou (2015) | Patient and public representatives | 32 | 81 | Patient organisations | Email invite sent | 24 overall – not broken down to panel (Algeria, Australia, Austria, Bahrain, Belgium, Brazil, Canada,  Colombia, Egypt, France, Germany, Greece, India,  Italy, Japan, Mexico, Morocco, the Netherlands, Saudi  Arabia, Spain, Taiwan, Tunisia, United Arab Emirates,  United Kingdom and United States of America) |
| Helliwell (2016) | Patient and public representatives | 55 | 84 | Not clear | Not clear | 1 (UK) |
| DM1 | Patient and public representatives | 30 | 70 | Not available | Not available | International*^~^* |
| DM2 | Patient and public representatives | 41 | 93 | Not available | Not available | International*^~^* |
| DM3 | Patient and public representatives | 15 | 67 | Not available | Not available | 1 (UK) *^~^* |
| DM3 | Patient and public representatives | 4 | 75 | Not available | Not available | 1 (UK) *^~^* |
| DM4 | Patient and public representatives | 7 | 43 | Not available | Not available | International*^~^* |
| DM4 | Patient and public representatives | 238 | 24 | Not available | Not available | International*^~^* |
| DM5 | Patient and public representatives | 67 | 75 | Not available | Not available | International*^~^* |
| DM6 | Patient and public representatives | 359 | 84 | Not available | Not available | International*^~^* |
| DM7 | Patient and public representatives | 18 | 89 | Not available | Not available | International*^~^* |
| Chiarotto (2015) | Mixed | 261 | 50 | Publications Known to steering committee | Invitations for participation were sent by email | 15 (US, The Netherlands, Australia, UK, Brazil, Italy, Norway, Canada, Spain, Belgium, Germany, Denmark, France, Finland, Switzerland) |
| Haeusler (2015) | Mixed | 43 | 86 | Publications  Clinical practice guideline panel | E-mail invitations from the steering group | 19 (not stated) |
| Eleftheriadou (2015) | Mixed | 18 | 94 | Professional organisations | Email invite sent | 24 overall – not broken down to panel (Algeria, Australia, Austria, Bahrain, Belgium, Brazil, Canada,  Colombia, Egypt, France, Germany, Greece, India,  Italy, Japan, Mexico, Morocco, the Netherlands, Saudi  Arabia, Spain, Taiwan, Tunisia, United Arab Emirates,  United Kingdom and United States of America) |
| Buch (2014) | Mixed | 21 | 100 | Not clear | Not clear | 12 (not stated)  *10 European countries, the US and*  *Canada* |
| Ismail (2016) | Mixed | 65 | 86 | Professional organisation | Potential participants were invited to take part by dissemination of an e-mail invitation and were asked to respond directly to the lead researcher | Not reported (international) |
| Fair (2016) | Mixed | 117 | 80 | Research consortium Conference participants | Survey distributed to the entire mailing list using a web-based platform | Not reported (international)  *US and other* |
| Major (2016) | Mixed | 10 | 100 | Publications | Not clear | 7 (Australia, Belgium, Canada, Netherlands, South Africa, UK, US) |
| DM1 | Mixed | 21 | 71 | Not available | Not available | International*^~^* |
| van ʼt Hooft (2015) | Researcher | 39 | 90 | Professional organisations | A formal written invitation was e-mailed to all members | International*^~^* |
| DM3 | Researcher | 8 | 88 | Not available | Not available | 1 (UK) *^~^* |
| DM4 | Researcher | 35 | 74 | Not available | Not available | International*^~^* |
| DM6 | Researcher | 92 | 93 | Not available | Not available | International*^~^* |
| DM7 | Researcher | 35 | 100 | Not available | Not available | International*^~^* |
| DM7 | Funder | 4 | 100 | Not available | Not available | 1 (US) *^~^* |
| DM6 | Commercial Representative | 43 | 86 | Not available | Not available | International*^~^* |

*^~^ Confirmed/provided through personal communication with the author.*
